# Stereolithographic Additive Manufacturing of High Precision Glass Ceramic Parts

**DOI:** 10.3390/ma13071492

**Published:** 2020-03-25

**Authors:** Julia Anna Schönherr, Sonja Baumgartner, Malte Hartmann, Jürgen Stampfl

**Affiliations:** Christian Doppler Laboratory for Photopolymers in Digital and Restorative Dentistry, TU Wien, Getreidemarkt 9, 1060 Vienna, Austria; sonja.baumgartner@tuwien.ac.at (S.B.); malte.hartmann@tuwien.ac.at (M.H.); juergen.stampfl@tuwien.ac.at (J.S.)

**Keywords:** biomedical engineering, additive manufacturing, stereolithography, micro CT, glass ceramic, dental replacement

## Abstract

Lithography based additive manufacturing (AM) is one of the most established and widely used 3D-printing processes. It has enabled the processing of many different materials from thermoplast-like polymers to ceramics that have outstanding feature resolutions and surface quality, with comparable properties of traditional materials. This work focuses on the processing of glass ceramics, which have high optical demands, precision and mechanical properties specifically suitable for dental replacements, such as crowns. Lithography-based ceramic manufacturing (LCM) has been chosen as the optimal manufacturing process where a light source with a defined wavelength is used to cure and structure ceramic filled photosensitive resins. In the case of glass ceramic powders, plastic flow during thermal processing might reduce the precision, as well as the commonly observed sintering shrinkage associated with the utilized temperature program. To reduce this problem, particular sinter structures have been developed to optimize the precision of 3D-printed glass ceramic crowns. To evaluate the precision of the final part, testing using digitizing methods from optical to tactile systems were utilized with the best results were obtained from micro computed tomography (CT) scanning. These methods resulted in an optimized process allowing for possible production of high precision molar crowns with dimensional accuracy and high reproducibility.

## 1. Introduction

Since the early 1990s additive manufacturing (AM) developed as a rapid manufacturing tool and today it competes with traditional manufacturing methods [1].AM is connected to digital tools such as 3D-scanning or computer tomographic imaging and enables the production of parts with highly complex geometries, which cannot be manufactured by conventional methods. AM is therefore an ideal manufacturing method for many applications in biomedical engineering, where individual, patient specific geometries or small-scale batches are required [2].

Dental restorations (crowns, bridges) or implants require high precision and outstanding mechanical properties, which can be obtained by AM. In particular, the marginal fitting accuracy is essential to ensure the needed aesthetic, and is essential to parodontal health and the longevity of the restauration [3,4]. In the case of ceramics, different AM procedures have been investigated regarding their suitability for manufacturing of ceramic parts: selective laser sintering (SLS), fused deposition modelling (FDM), 3D-printing and stereolithography (SLA) [2,5,6,7]. SLA-based ceramic manufacturing also called lithography-based ceramic manufacturing (LCM) was the selected process for this study [8]. LCM enables the processing of highly filled and photopolymerizable ceramic slurries, which allows for the production of fully dense ceramic parts with outstanding material properties. This methodology was also tested for different ceramic compositions including alumina (Al_2_O_3_), tricalcium phosphate (Ca_3_(PO_4_)_2_) and lithium disilicate (Li_2_Si_2_O_5_) (see Table 1).

Furthermore, LCM provides the best resolution when compared with other binder-based methods [14]. In this study, the process is optimized for the development of high precision ceramic parts. The LCM-process includes a number of different steps, each of which has its influence on the precision of the final product. First of all, a slurry had to be developed and adjusted to the particular AM process. After data preparation, the three-dimensional composite structure, the so-called green body, is then built layer by layer in the machine [15]. After cleaning and removing support structures, the thermal post processing starts with the debinding of the green body followed by the dense sintering. The final stage is post processing which includes several different steps, such as removing of sinter support structures, polishing and glazing, depending on the application [8].

To analyze the accuracy of a three-dimensional ceramic part several methods are available. While one of the first established scanner systems for dental applications was the touch-probe scanning based Procera system (Nobel Biocare AB, Gothenburg, Sweden) [16], this study uses different scanning methods, such as optical and tactile scanners, as well as micro-CT, to determine which is the most suitable [17].

## 2. Materials and Methods 

### 2.1. Lithograpy-Based Ceramic Manufacturing

There are four different steps in the lithography-based AM of ceramic parts: (1) the digital data preparation; (2) the slurry development; (3) the initial printing process and; (4) the post processing which is the thermal treatment. The essential process steps (printing and thermal treatment) are called lithography based ceramic manufacturing (LCM). This paper addresses the resolution of LCM processed ceramic parts and focuses on the process of optimized part accuracy. The base of the slurry contains a variety of monomer compositions and solvents as well as a photo absorber, a photo initiator and the ceramic filler with a solid loading of more than 50 vol %. The glass ceramic filler is Li_2_O/SiO_2_ with an average grain size of 6 µm and a refractive index of 1.53. The content ratios are given in Table 2. 

After producing the shape, known as a green body, using the LCM process, the residual slurry adhering to the green body has to be removed. This is achieved by cleaning the samples using a soft waterjet. Thermal treatment follows with the removal of organic solvent and binder material using a thermal program up to 400 °C, then sintering to full density up to 850 °C.

**Figure 1:**
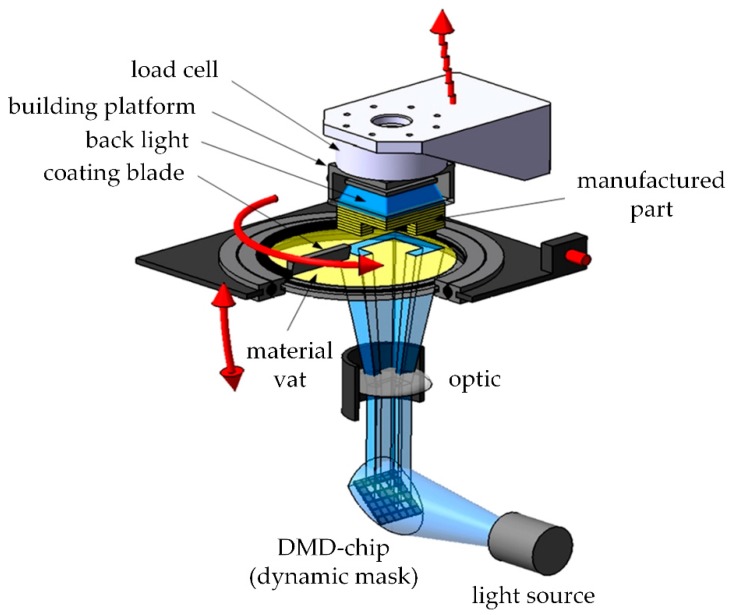
DLP system, schematic setup (modified [19]).

The digital light processing (DLP) -based system (shown in Figure 1) uses a LED-light source in combination with a dynamic mask to cure every single layer. The stereolithographic process bases on a light source, which cures a photosensitive resin through a transparent material vat (glass with a top layer of silicon). The first layer sticks on the building platform and upwards. After the application of new material, using a coating blade, the process is repeated [18]. The precision in x and y direction is strongly dependent on the resolution of the digital micro mirror device (DMD). However, it changes with the wavelength of the light source. For this study, a DMD with a pixel size of 25 × 25 µm² and a LED with a wavelength of 460 nm was used. The precision in z-direction is dependent on the penetration depth of light in the material. Based on this, the selected layer height of 25 µm was chosen.

### 2.2. Support Structures

The initial digital modelling and design of support structures influences the final precision of the part. The support structures have two functions in the case of ceramics in that they enable the structuring of the part in the printer (especially for overhanging areas) and, can assist in reducing sinter warping. Additionally, it is important to find an optimal orientation of the part with respect to these support structures.

A molar crown, which was available as an stl file (extract from micro-CT scan), was chosen for this study and all the samples have been developed out of it. The method to create a suitable support is explained using this molar crown as an example.

**Figure 2:**
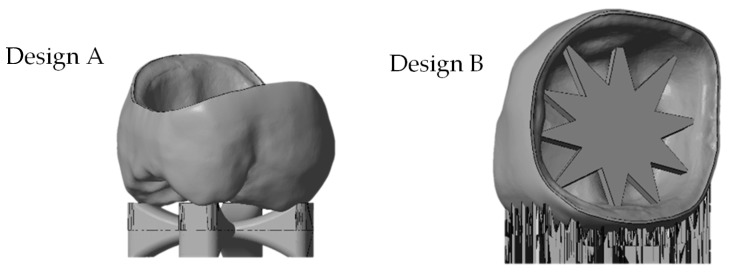
stl file of the molar crown with different support designs (from left: ‘cross’-support Design A, ‘star’-support Design B).

Two design elements were developed to control the shrinkage while sintering to reduce the warpage. A ‘cross’-support was designed on the occlusal side of the crown (see Figure 2, Design A). An adjusted star structure was also developed (see Figure 2, Design B), which led to an additional support structure on the oral side to make the building process possible. 

It should be noted that support structures are necessary for both the precision and position of the final product. Analysis shows that the following areas of the crown should be avoided for support placement [4]:

1. cusps
2. distal and mesial surfaces
3. gap between crown and core (inner surface).

**Figure 3:**
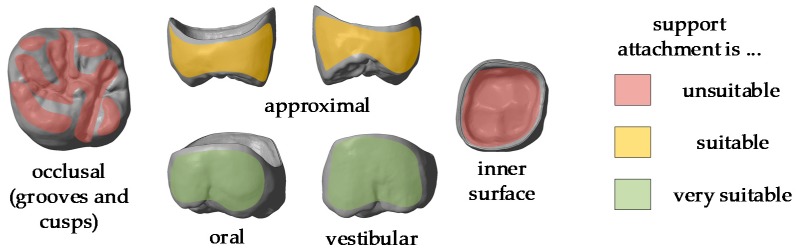
stl-file of a molar crown with marking the different areas of support attachment.

Figure 3 shows the stl file of the printed crown. The most critical areas to be avoided are marked in red, the intermediate ones in yellow and the most suitable areas for support attachments are marked in green.

**Figure 4:**
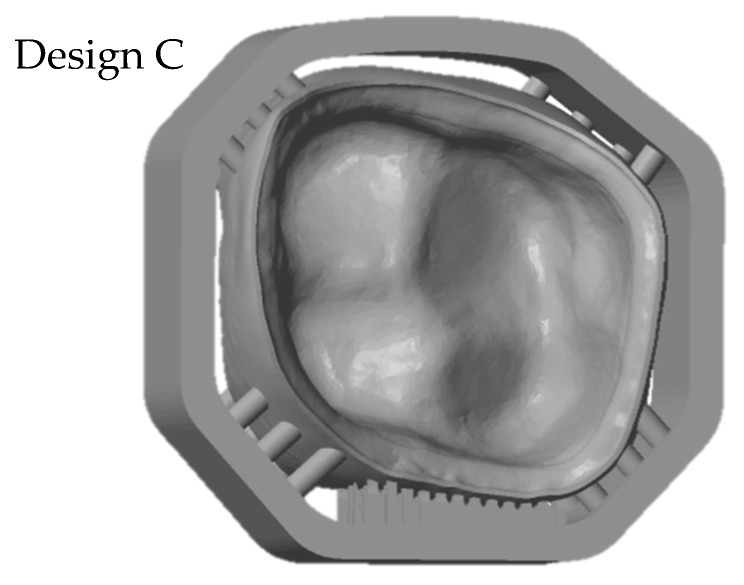
stl-file of the molar crown with optimized support design (‘milling’ support, Design C).

To fulfil all these requirements the support design was redeveloped. The so called ‘milling’ support has a frame with pins to support the crown. These supports do not attach either the occlusal side or the inner surface (see Figure 4, Design C).

### 2.3. Digitizing Methods

To determine which design led to the most precise crown, the finished part was digitized. The resulting digital version was compared to the original stl file. Various methods of digitizing a glass ceramic part were assessed to ascertain which has the best accuracy. The samples were digitized using laser scanner, 3D light microscope, medical micro-CT and tactile scanner. The red laser scanner D810 by 3Shape (3Shape A/S, Copenhagen Denmark) has a precision of 15 µm. The model was placed on a table in the scanner where the laser projected lines on the molar crown, which was then photographed via high resolution digital cameras. The table where the model is located can be rotated and tilted as well as moved on a horizontal axis to make sure the complete surface can be scanned. 3Shape provides a software which calculates a point cloud as a 3D model by triangulation.

A second contactless method to digitize the sample utilized an Alicona Infinite Focus Microscope (Alicona Imaging GmbH, Graz, Austria). The principle of measurement is optical microscopy and focus variation technology to transfer 3D morphology and depth information from the surface into a digital 3D model. The underlying accuracy amounts to 8 µm. 

To make sure there are no optical influences the tactile measurement Renishaw Cyclone 2 (Renishaw, Pliezhausen, Germany) was used. The resolution of the used setup is 15 µm. The model has been scanned with a stylus diameter of 1 mm and a row width of 50 µm.

The last used method is the MicroCT100 from Scanco (Scanco Medical AG, Brüttisellen, Switzerland) operating at 70 kV, 200 µA and 14 W with a voxel size of 10 × 10 × 10 µm³. Using the micro-CT Evaluation Program (V6.5-3, Scanco Medical AG, Brüttisellen, Switzerland) the raw data have been segmented and subsequently converted into stl files.

## 3. Results and Discussion

### 3.1. Resolution Tests

**Figure 5:**
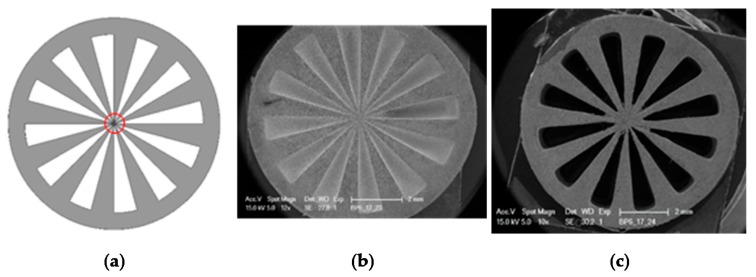
Siemens star, diameter 9 mm: (**a**) stl file used for the study; (**b**,**c**) SEM images of green bodies.

One challenge of lithography-based processing of ceramic slurries is the scattering of light because it produces lower resolution green body structures. An optimal light absorbing agent with an absorbance maximum at the same wavelength as the light source was used to minimize this phenomenon. To ensure that the correct absorber was selected, value resolution tests were conducted and different test structures were printed. To understand the resolution of the filter the so called ‘siemens star’ was printed with various parameters. The siemens star structure was developed by Werner von Siemens in the 1930s to get information about the quality of cinema cameras. The star structure forms a point in the center whose size is dependent on the resolution [20] (see red circle in Figure 5).

Using this star structure, the content of the light absorber was adjusted as well as the printing parameters (intensity and exposure duration). Additionally, the resolution could further be improved by changing the wet layer thickness in the material vat of the printer from 220 to 170 µm (see Figure 5b compared to c). The resulting structure (green body) is shown in Figure 5c. The open structures are completely free, resulting in a small inner circle of around 1mm.

### 3.2. Scaling Factor

**Figure 6:**
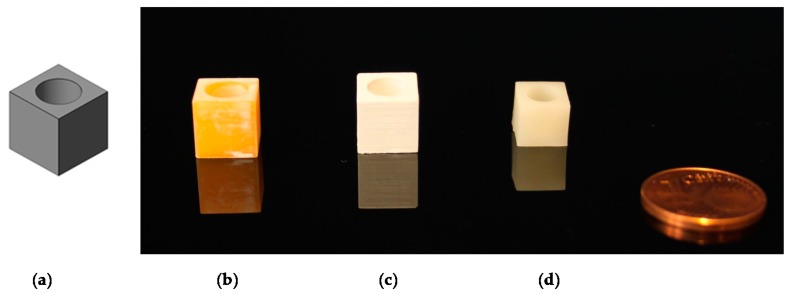
Scaling cubes: (**a)** stl file; (**b**) green body; (**c**) debinded body; (**d**) sintered body.

Another key task is the evaluation of the scaling factor due to sinter shrinkage of around 25 percent in every direction. To test this, ten cubes with a side length of 9 mm and a cylindrical hole with a depth of 8 mm and a diameter of 8 mm were produced using the LCM process (see Figure 6).

The sintered cubes were measured, and the shrinkage factor (approximately 20% in every direction) was included in the file preparation for digital upscaling of the green body.

### 3.3. Comparison of Different Scan Methods

To compare the different methods one crown, with the ‘star’ support structure (‘Design B’) was processed with the LCM method and digitized by all systems. As shown in Table 3, the different methods produced various results. There were challenges in reproducing the real surface due to reflections and translucencies of the ceramic, which resulted in the appearance of a so called ‘orange skin’ effect using the 3Shape D810 infrared scanner. This system was developed to scan cast models for dental applications and is not optimized for glass ceramics. The next method is also based on a contactless light scanning system. The optical microscope Alicona Infinite Focus G5 scans the part using different focus planes in the z direction. It also does not give optimal results due to the translucent and reflective surfaces, which lead to artefacts, and hence an incorrect reproduction of the surface (see Detail B). The result, shown in Table 3, is more precise than 3Shape D810 scanner but nevertheless it is obvious that details are missing when compared to the Reinshaw Cyclone and micro-CT results. The third technology is a tactile measurement. The resolution used for this setup is 15 µm. Detail A shows the oral surface of the crown where the resolution of the scanning method can be evaluated. However, the method is comparative slower, sensitive towards distinct surface structures and not state of the art [21]. The original file has to be approximated by slicing the stl file into the single layers. This approximated surface is by the last scanning method the MicroCT100 from Scanco. Moreover, the micro-CT scan point details can be seen clearly as illustrated by the small chip along the edge of the crown (also seen in Detail A). However, one shortcoming in evaluation methods is the fact that ceramics, and especially glass ceramics, are often more or less translucent and have a reflective surface; therefore micro-CT scan is the most suitable digitizing method.

### 3.4. Comparison of Different Support Designs

The LCM processed crowns were compared with the original stl file. For comparison, the stl was rescaled with the calculated factors in every direction (x, y and z). After printing the parts using the SLA process they were cleaned, and all the support structures remained on the crowns. The cleaning was achieved using a soft waterjet (solution of water and 0.5% detergent TeepolTM 610 S). Additionally, the thermal post processing took part in the same orientation as the building process. Since the best results were obtained from micro-CT scans, this was the selected method for subsequent analysis. The precision of the LCM processed crowns was evaluated by overlaying the stl file with the scan file and applying a geometric tolerances analysis, based on GOM Inspect (GOM GmbH, Braunschweig, Germany). To simplify the procedure, only the inner surface of the crown was analyzed. 

**Figure 7:**
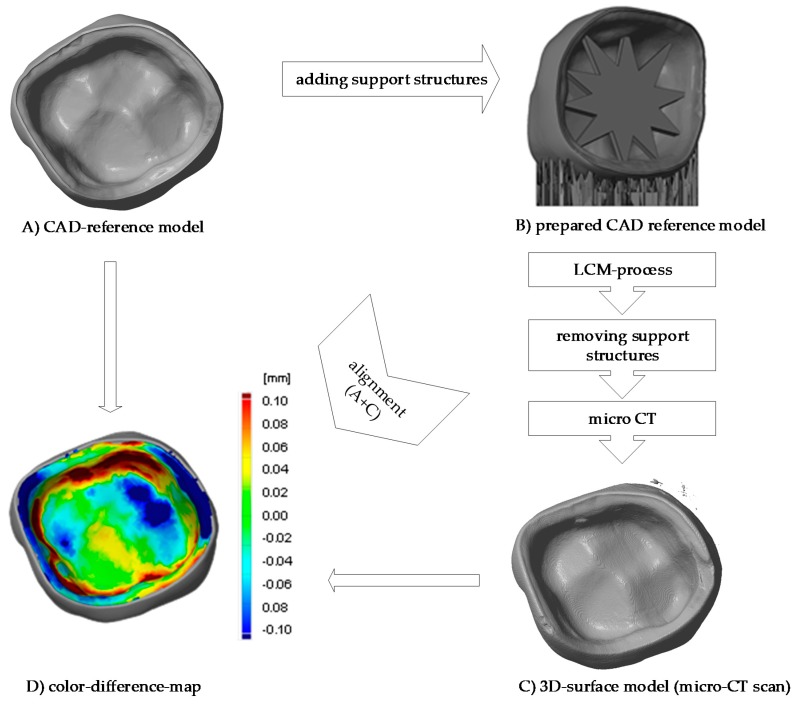
Procedure of evaluating the precision of the 3D-printed crown (**a**) CAD-reference model, (**b**) prepared CAD reference model, (**c**) micro-CT scan of 3D-printed and sintered molar crown, ‘star’ support was removed after sintering (**d**) comparison of CAD-reference model and 3D-surface-model using GOM Inspect.

All samples were processed using a similar methodology, with the only difference being the design of the support structures. The crown with a ‘star’ support at the inner surface and the one with building support on the oral surface were compared (Figure 7). The star support was removed with a dental drill after sintering. The dark red color shows that by removing the star too much material was lost, resulting in poor precision at the inner surface with deviations of more than 100 µm leading to a lower quality of precision in an essential area. The dark blue color (deviation of minus 100 µm), especially at the edges shows, that the crown warps during sintering. The star therefore does not provide enough support.

**Figure 8:**
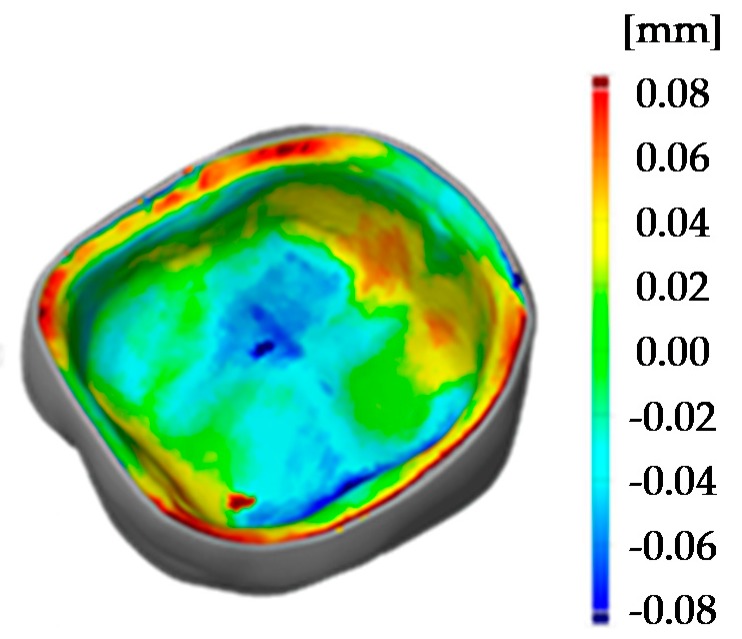
Comparison of 3D surface model (sintered molar crown with ‘milling’ support, design C) and stl file using GOM Inspect.

To reduce the sinter warpage and in order to not interfere with essential surfaces of the crown, the support design was redeveloped. Molding sprues were used to create a support structure around the crown like a frame with only little pins holding and supporting the crown (see Figure 4, design C). All support structures remained on the crown while scanning. The benefit of this design is seen in the Figure 8 (design C), where the printed crown is compared to the stl file. The improved support design enables a remarkable increase of precision, especially since the edges are no longer warped. Ultimately, the range of the color scale could be reduced to minus 80 µm to plus 80 µm. Most parts of the crown have a deviation around ±20 µm.

**Figure 9:**
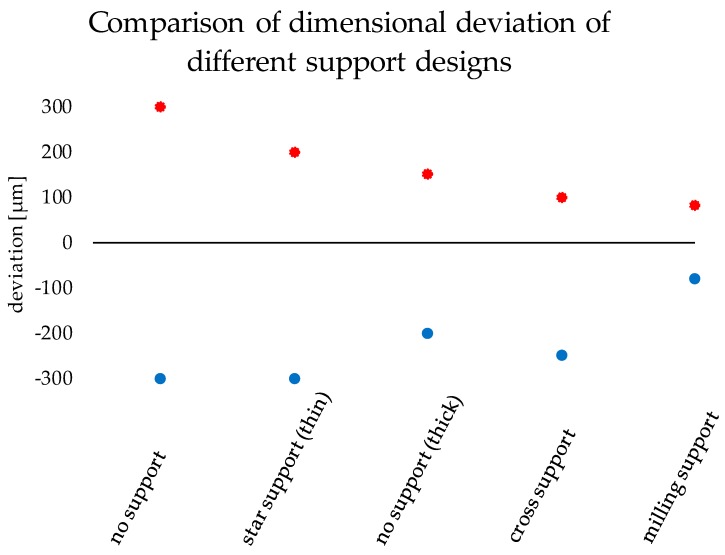
Comparison of dimensional deviation of the different support designs.

The improvement of the dimensional accuracy can be seen by using statistical information to compare all tests support types (Figure 9). This was achieved by laying all analyzed crowns on top of the same original file and a surface comparison was done. After, the deviation scale was adjusted to highlight only relevant measurements and that all the colors in the false color diagram were represented. Finally, the maximal and minimal deviation of the color false scale is used for measuring scale in Figure 9.

**Figure 10:**
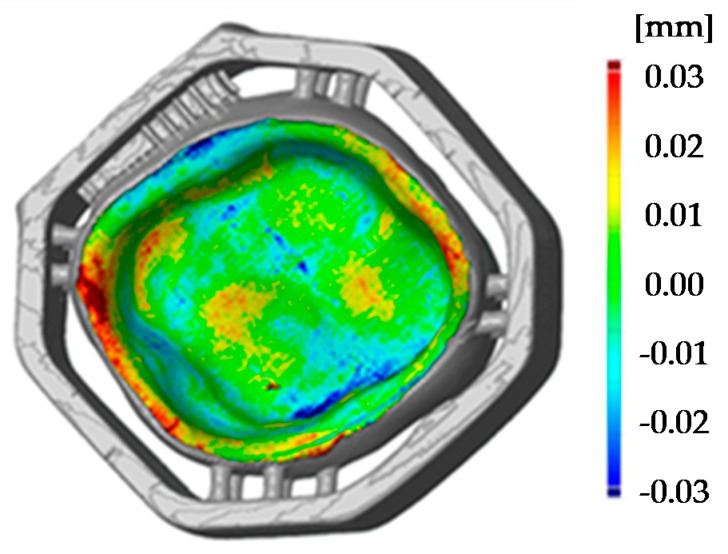
Comparison of two 3D surface models (sintered molar crown with ‘milling’ support, design C).

To show the reproducibility of the process two LCM processed crowns were compared as illustrated in the pseudo color image in Figure 10. The maximum deviation amounts to 30 µm, which shows a high reproducibility of the whole process chain. These tolerances are also sufficiently low for allowing clinical use of such crowns. The maximum tolerance accepted for clinical use has been discussed during the years and is defined between 50 to 120 µm [3,4,20,21].

## 4. Conclusions

**Figure 11:**
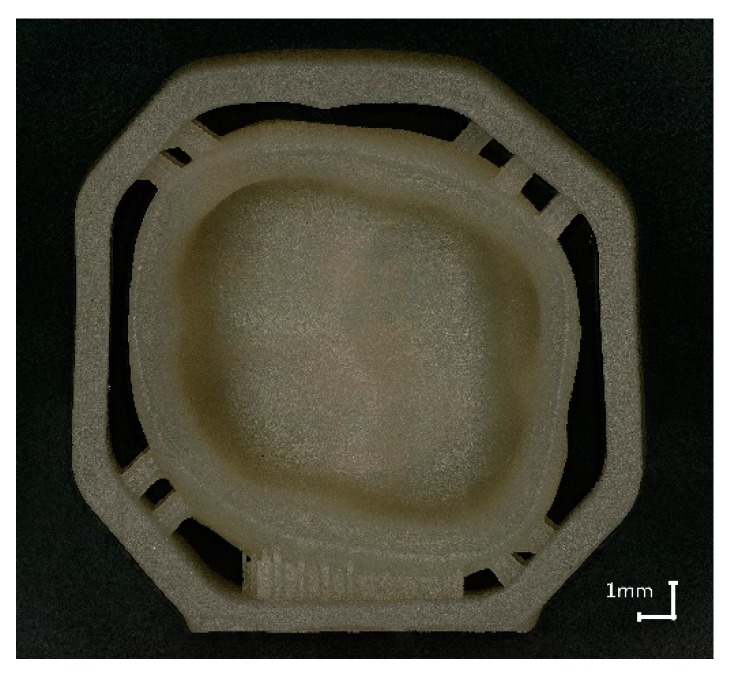
Molar crown with ‘milling’ support, additively manufactured, sintered.

The aim of this study was to optimize the precision of glass ceramic additively manufactured parts, especially dental molar crowns. The precision of the glass ceramic parts was tested using tactile and optical methods, with the best results being obtained by micro CT scan, since it allowed the continual assessment of the process and optimizing the processing of the parts. Sinter structures were developed to reduce the warpage while sintering and the maximum deviation (manufactured part compared to the digital one) of ±300 µm could be reduced to ±80 µm. The most effective one is the ‘milling’ support which surrounds the molar crown like a cage as seen in Figure 11 (additively manufactured green body).

**Figure 12:**
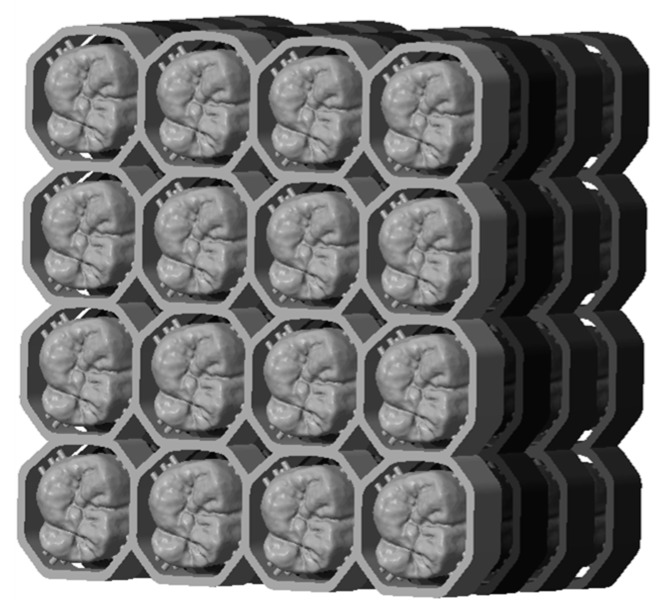
‘Milling’ support enables stacking and stringing together.

If we look to the future the ‘milling’ support can be in addition a useful supplement as it enables a simplified handling, by machining ‘pick and place’. Additionally, it could also be used to mark the single parts with QR tags or numbers, which is helpful especially by printing many crowns in one step. Due to the structures, the parts are stackable and volume can be built with a printer, this process can be optimized for manufacturing exploit (see Figure 12) [3].

By comparing two processed crowns using surface analyses of digital data showed the high precision of the crowns (±80 µm) and high reproducibility (±30 µm maximum deviation).

## 5. Patents

One patent result from the work: Schönherr, J.; Ebert, J.; Gmeiner, R. Verfahren zur additiven Fertigung von Formkörpern, 03-12-2018. EP18209713.

## Figures and Tables

**Table 1:** Material properties of different ceramics achieved with lithography-based ceramic manufacturing (LCM) [1,9,10,11,12,13].

	Material				
	Al_2_O_3_	ZrO_2_	45S5 Bioglass^®^	Ca_3_(PO_4_)_2_	Li_2_Si_2_O_5_
Measured biaxial bending strength (MPa)	521	1098	124	32	344.5
Biaxial bending strength in literature (MPa)	300–580	1000	42	24	215–400
Density (g/cm^3^)	3.82	5.9	2.7	3.14	2.508
Relative Density (%)	99.6	99.92	>99	88	>99.9
Solid loading—green parts (%)	50	42	47.7	50	54.3

**Table 2:** The main components of the slurry.

Component	wt %
Bifunctional methacrylate	7.3
Trifunctional acrylate	9.2
Photo initiator	0.05
Light absorber	0.0025
Solvent	9.445
Dispersing agent	1
Glass ceramic powder	73.0025

**Table 3:** Images of crown A obtained with different scan methods.

Method	Image	Detail A	Detail B
3Shape D810	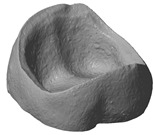	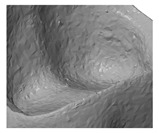	---
Alicone Infinite Focus G5	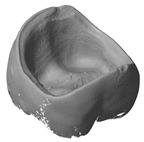	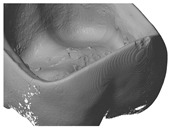	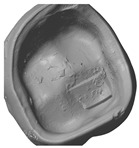
Renishaw Cyclone 2 Tactile measurement	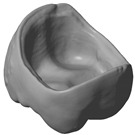	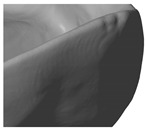	---
Scanco Micro CT 100	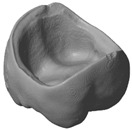	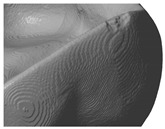	---
